# Exogenous Melatonin Regulates the Flavonoid Biosynthesis Pathway to Alleviate Saline–Alkali Stress in *Ulmus pumila* ‘Zhonghua Jinye’

**DOI:** 10.3390/plants15131960

**Published:** 2026-06-25

**Authors:** Songhua Dai, Yichao Liu, Shufang Yan, Yinran Huang, Shuxiang Feng, Guojun Zhang

**Affiliations:** 1 College of Horticulture Technology, Hebei Normal University of Science and Technology, Qinhuangdao 066000, China; 13331377321@189.cn; 2 Hebei Academy of Forestry and Grassland Science, Shijiazhuang 050061, China; 15830991106@163.com (Y.L.); 15373818588@163.com (S.Y.); 3 College of Landscape and Tourism, Hebei Agricultural University, Baoding 071000, China; 18832263768@163.com

**Keywords:** *Ulmus pumila* ‘Zhonghua Jinye’, melatonin, saline–alkali stress, flavonoid biosynthesis, transcriptome, metabolome

## Abstract

Melatonin, a potent endogenous antioxidant, holds promise for enhancing stress tolerance in woody plants, yet its molecular mechanism under saline–alkali stress remains poorly understood. This study systematically investigated the effects of exogenous melatonin on *Ulmus pumila* ‘Zhonghua Jinye’ by integrating physiological assays, transcriptomics, and metabolomics. Two-year-old cuttings were subjected to 150 mmol·L^−1^ saline–alkali stress and treated with varying melatonin concentrations (0, 50, 100, 200, 400 μmol·L^−1^; three replicates). Physiological evaluations identified 100 μmol·L^−1^ melatonin (SMT100) as optimal, significantly enhancing antioxidant enzyme activities (SOD, CAT, APX, GR) by 28.7–41.5% and reducing reactive oxygen species (H_2_O_2_ by 31.5%; O_2^−^_ by 38.2%) compared to untreated stressed controls. Integrated omics analysis (CK, S, SMT100 groups) revealed that saline–alkali stress suppressed the flavonoid biosynthesis pathway, down-regulating key genes such as *UpANS1* (10.74-fold), *UpANS2*, *UpHCT1*, and *UpDFR2*, thereby reducing the accumulation of protective flavonoids like quercetin and kaempferol. Conversely, melatonin treatment reactivated this pathway, significantly up-regulating *UpANS1* (17.36-fold induction), *UpDFR2* (5.55-fold), *UpCHS1*, *UpF3H6*, and *UpLAR2*. This genetic reconfiguration promoted the synthesis of antioxidant flavonoids, enhancing the plant’s overall stress resilience, thus identifying *UpANS1* as candidates associated with treatment response. The study provides a scientific basis for cultivating *U. pumila* ‘Zhonghua Jinye’ in saline–alkali soils and clarifies the molecular mechanism by which melatonin alleviates combined saline–alkali stress via flavonoid pathway regulation.

## 1. Introduction

Soil salinization represents a significant threat to ecological security and hinders the progress toward achieving the Sustainable Development Goals [1]. Currently, there are about 9.9 × 10^7^ hm^2^ of salt-affected soils in China, in which modern saline soil is about 3.7 × 10^7^ hm^2^ [2]. High concentrations of salt ions—such as Na^+^, Cl^−^, CO_3_^2−^, and HCO_3^−^_—can induce osmotic stress, ion toxicity, nutrient imbalance, and oxidative damage in plants, severely restricting their growth and development [3]. For example, under combined saline–alkali stress, both OT hybrid lily and Caragana seedlings exhibited physiological damage and growth inhibition: in OT hybrid lily, relative electrolyte leakage and malondialdehyde (MDA) content increased under stress, while root activity and soluble protein content decreased when the stress concentration exceeded a certain threshold [4]; in Caragana seedlings, the stress significantly reduced biomass accumulation and growth rates, altered the root-to-shoot ratio, decreased photosynthetic pigment content and PSII activity, inhibited antioxidant enzyme activities, and aggravated membrane lipid peroxidation damage [5]. Therefore, improving plant tolerance to saline–alkali stress is crucial for the biological remediation and sustainable utilization of saline–alkali soils [6,7].

*Ulmus pumila* ‘Zhonghua Jinye’ (genus *Ulmus*, family Ulmaceae) is an excellent ornamental tree species valued for its golden-yellow foliage, extended ornamental period, and strong adaptability. It is widely used in urban landscaping and ecological engineering projects [8]. However, saline–alkali stress can lead to leaf wilting, marginal scorching, and even plant death, substantially limiting its widespread application in many saline–alkali regions [9]. Investigating the saline–alkali tolerance potential of *U. pumila* ‘Zhonghua Jinye’ and elucidating its underlying regulatory mechanisms are of considerable practical importance for expanding its adaptability and supporting greening initiatives in saline–alkali areas.

Melatonin, a potent free radical scavenger and antioxidant, also functions as an important signaling molecule involved in regulating plant growth and development, photoperiod responses, and various abiotic stress responses [10]. Numerous studies have demonstrated that exogenous melatonin application can effectively alleviate plant damage caused by stresses such as salinity, drought, low temperature, and heavy metals [11]. Its mechanisms of action operate at multiple levels, including enhancing antioxidant enzyme activity, regulating ion homeostasis, maintaining photosynthetic apparatus function, inducing the expression of stress-related genes, and interacting with other hormone signaling pathways [12,13]. Under saline–alkali stress, melatonin has been shown to improve tolerance in crops such as rice, wheat, tomato, and cucumber by regulating Na^+^/K^+^ balance, promoting the accumulation of compatible solutes, and activating antioxidant defense systems [14]. However, existing research has predominantly focused on herbaceous model plants and crops. Studies on melatonin in woody plants, particularly regarding its role in regulating secondary metabolic pathways under combined saline–alkali stress, remain limited [15].

Flavonoids are an important class of polyphenolic secondary metabolites in plants, possessing diverse physiological functions such as antioxidant activity, UV protection, antibacterial properties, and roles in signal transduction [16,17]. Their biosynthesis begins with phenylalanine, which is subsequently converted through the phenylpropanoid pathway and the flavonoid pathway via a series of enzymatic reactions, yielding various compounds including flavanones, flavones, flavonols, and anthocyanins [18]. Research indicates that abiotic stresses such as drought, salinity, and UV radiation can significantly influence the expression of flavonoid biosynthesis-related genes and the accumulation of corresponding metabolites, thereby modulating plant stress resistance [19]. Recent studies have shown that melatonin can also affect flavonoid metabolism in plants. For instance, melatonin treatment has been reported to increase flavonoid content in soybean and grape, and to enhance plant antioxidant capacity by regulating the expression of related genes [20,21]. However, the specific molecular players—particularly the key structural genes (e.g., *ANS*, *DFR*) and transcription factors within the flavonoid pathway—that respond to melatonin in woody plants like *U. pumila* ‘Zhonghua Jinye’ remain unidentified. Furthermore, while melatonin is known to alter flavonoid content, the simultaneous dynamic changes in gene expression and metabolite profiles in this specific cultivar under combined saline–alkali stress have not been reported.

Based on this research background, the present study used *U*. *pumila* ‘Zhonghua Jinye’ as experimental material with the following objectives: (1) to clarify the ameliorative effects of exogenous melatonin on the physiological characteristics of golden-leaf elm under saline–alkali stress and to determine its optimal application concentration; (2) to reveal the molecular response features of melatonin-treated golden-leaf elm at both the transcriptional and targeted metabolite accumulation levels; and (3) to identify candidate genes and pathways involved in the melatonin-mediated mitigation of saline–alkali stress using integrated multi-omics analysis.

Ultimately, this study systematically elucidates the molecular mechanism by which melatonin enhances the saline–alkali tolerance of *U. pumila* ‘Zhonghua Jinye’ via the regulation of the flavonoid biosynthesis pathway. The findings are expected to provide new insights into the role of melatonin in the stress resistance of woody plants and to support the genetic improvement of salt-tolerant forest trees.

## 2. Results

### 2.1. Effects of Exogenous Melatonin on Antioxidant Enzyme Activities and Reactive Oxygen Species in U. pumila ‘Zhonghua Jinye’ Under Saline–Alkali Stress

Under 150 mmol·L^−1^ saline–alkali stress, leaves of *U. pumila* ‘Zhonghua Jinye’ were sprayed with melatonin at different concentrations (0, 50, 100, 200, and 400 μmol·L^−1^). The results showed that 100 μmol·L^−1^ melatonin treatment (SMT100) exhibited the most significant alleviatory effect on stress injury.

**Figure 1:**
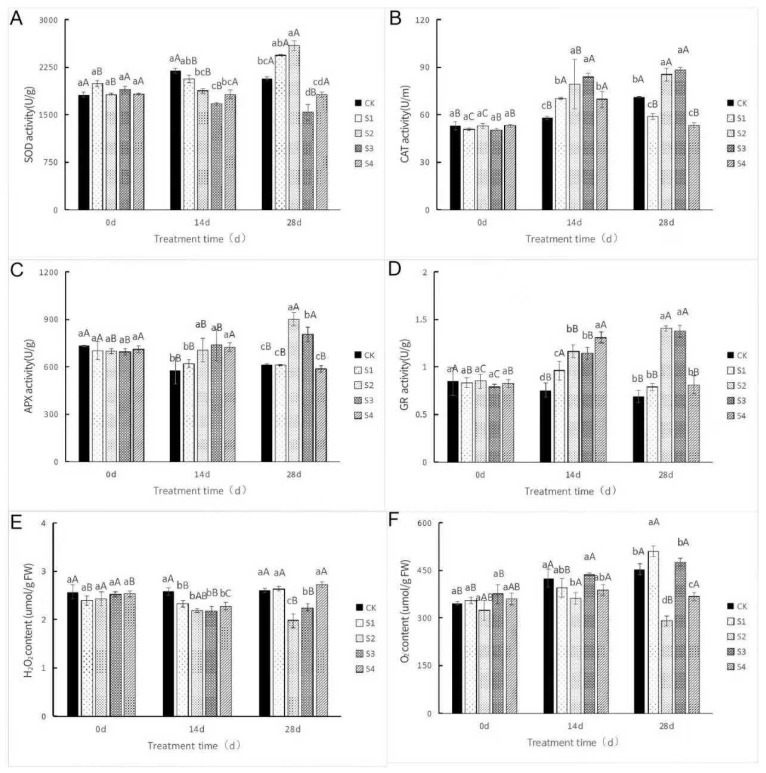
Effects of exogenous melatonin on antioxidant enzyme activities and reactive oxygen species in *U. pumila* ‘Zhonghua Jinye’ under saline–alkali stress. Note: (**A**) Superoxide dismutase activity; (**B**) Catalase activity; (**C**) Ascorbate peroxidase activity; (**D**) Glutathione reductase activity; (**E**) H_2_O_2_ content; (**F**) O_2^−^_ content. Different lowercase letters indicated that there was a significant difference in content under different MT concentrations at the same time (*p* < 0.05), and different uppercase letters indicated (*p* < 0.01).

Compared with the S treatment, SMT100 significantly increased the activities of SOD, CAT, APX, and GR in leaves at the late stress stage by 32.1%, 28.7%, 41.5%, and 25.6%, respectively (Figure 1A–D). Meanwhile, SMT100 effectively alleviated oxidative damage; the contents of H_2_O_2_ and O_2^−^_ were decreased by 31.5% and 38.2%, respectively, compared with the S treatment (Figure 1E,F).

Notably, the alleviating effect of exogenous melatonin on saline–alkali stress exhibited a significant concentration-dependent variation, rather than a continuous increasing trend with elevated concentrations. Integrated statistical comparisons among all five treatment groups revealed that low-concentration melatonin (SMT50) only slightly improved physiological indicators with limited mitigation effects, while moderate-concentration melatonin (SMT100) achieved the maximum enhancement of antioxidant enzyme activities and the minimum accumulation of reactive oxygen species. However, further increasing melatonin concentration to 200 μmol·L^−1^ (SMT200) gradually weakened the stress-alleviating efficacy, and the SMT400 treatment showed a significantly diminished protective effect compared with SMT100 (*p* < 0.05). Specifically, the SOD and CAT activities in the SMT400 group were decreased by 7.0% and 6.0%, respectively, relative to the SMT100 group, though these values were still markedly higher than those in the single saline–alkali stress group. Therefore, SMT100 was selected as the optimal treatment for subsequent molecular mechanism studies.

### 2.2. Transcriptome Sequencing and Differential Expression Analysis

**Figure 2:**
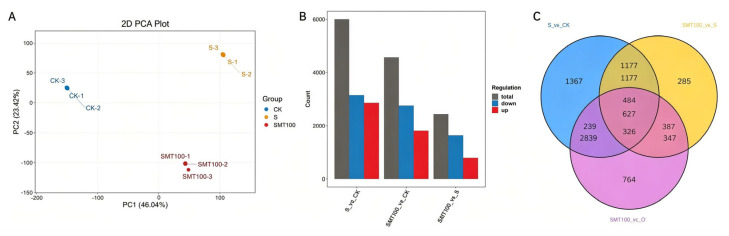
PCA chart and differential gene statistics chart. Note: (**A**) PCA chart; (**B**) differential gene statistics; (**C**) differential gene Venn diagram.

Transcriptome sequencing of nine samples was performed on the Illumina HiSeq™ 2500 platform (Illumina, San Diego, CA, USA). Summary statistics are listed in Table 1. A total of 75.39 Gb of clean data were generated, with at least 6 Gb of clean data obtained for each sample. The Q30 base ratio ranged from 95.84% to 97.43%, and the average GC content was 45.57%. Principal component analysis (PCA) was conducted to group biological replicates (Figure 2A). The variances of PC1 and PC2 were 46.04% and 23.42%, respectively. The saline–alkali stress group (S) was clearly separated from the control group (CK) along the PC1 axis, whereas the melatonin-treated group (SMT100) was distinctly distributed between the S and CK groups and shifted toward the CK group.

Differentially expressed genes (DEGs) were identified using DESeq2 with thresholds of |log_2_ Fold Change| > 1 and *p*adj < 0.05. Expression analysis of transcriptome data is shown in Figure 2B,C. In the S vs. CK comparison, 6010 DEGs were detected, including 2863 up-regulated and 3147 down-regulated genes. In the SMT100 vs. S comparison, 2436 DEGs were identified, consisting of 797 up-regulated and 1639 down-regulated genes. In the SMT100 vs. CK comparison, 4577 DEGs were found, with 1821 up-regulated and 2756 down-regulated genes.

### 2.3. Functional Enrichment Analysis of Differentially Expressed Genes

**Figure 3:**
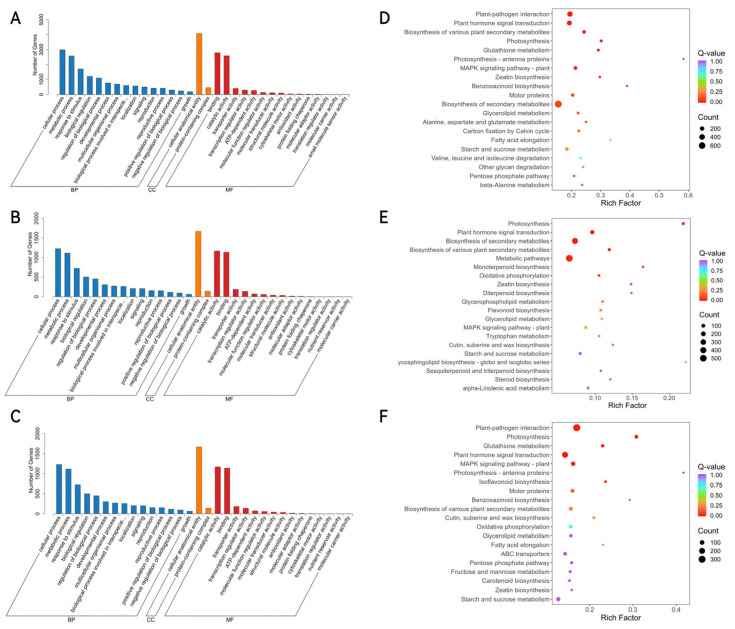
Differential gene GO enrichment map and differential gene KEGG enrichment scatter plot. Note: GO enrichment map: (**A**) S vs. CK; (**B**) SMT100 vs. S; (**C**) SMT100 vs. CK. KEGG enrichment: (**D**) S vs. CK; (**E**) SMT100 vs. S; (**F**) SMT100 vs. CK.

To further clarify the differences caused by exogenous melatonin treatment, functional enrichment analysis of DEGs was performed using the GO and KEGG databases. GO functional annotation of the assembled DEGs is shown in Figure 3A–C. DEGs were categorized into three main groups: biological process, cellular component, and molecular function. For biological processes, DEGs were significantly enriched in cellular metabolism, secondary metabolism, and response to stimuli. For cellular components, DEGs were mainly enriched in cellular responses and protein complexes. For molecular functions, DEGs were primarily enriched in DNA binding and catalytic activity. Molecular functions in the S vs. CK and SMT100 vs. CK groups changed more significantly than those in the SMT100 vs. S group.

**Figure 4:**
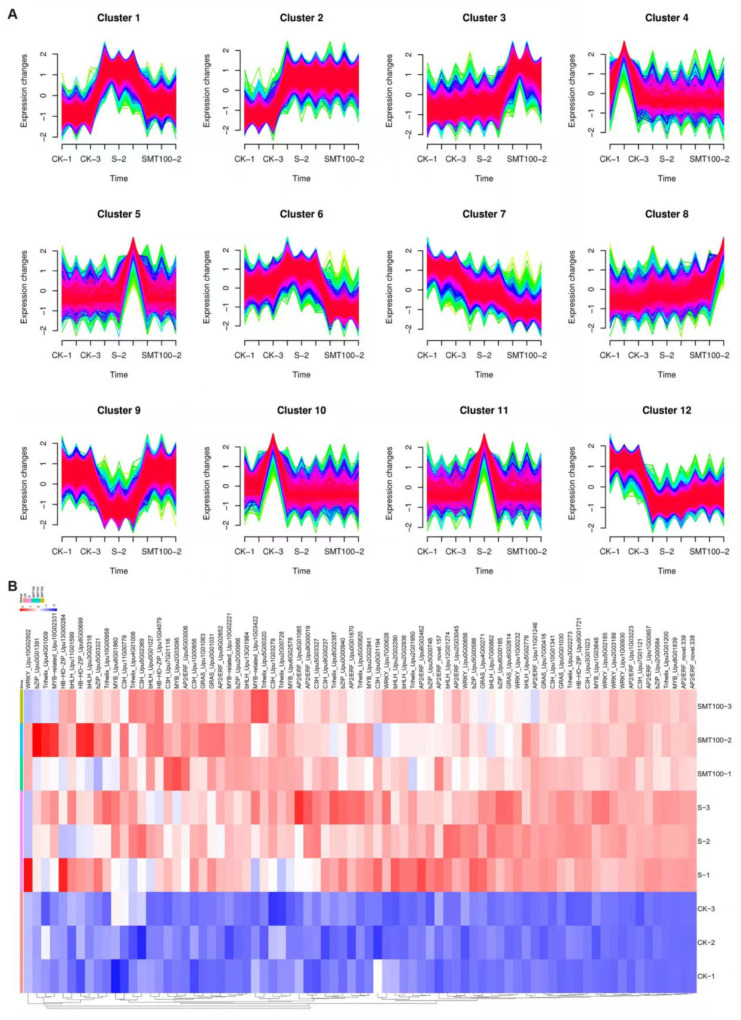
Gene expression pattern clustering heat map and transcription factor expression clustering heat map. Note: (**A**) Gene expression pattern clustering heat; (**B**) transcription factor expression clustering heat map.

The KEGG pathway enrichment analysis of DEGs is shown in Figure 4A,B. DEGs were mainly enriched in pathways including the biosynthesis and metabolism of secondary metabolites, antioxidant systems, photosynthesis, and flavonoid biosynthesis. In the S vs. CK group, DEGs were enriched in the biosynthesis of secondary metabolites, MAPK signaling pathway, plant hormone signal transduction, and flavonoid biosynthesis. In the SMT100 vs. S group, DEGs were enriched in the biosynthesis of secondary metabolites, plant hormone signal transduction, starch and sucrose metabolism, and flavonoid biosynthesis. In the SMT100 vs. CK group, DEGs were enriched in the biosynthesis of secondary metabolites, starch and sucrose metabolism, and flavonoid biosynthesis. Both saline–alkali stress and melatonin treatment affected DEGs involved in the biosynthesis of secondary metabolites and flavonoid biosynthesis, indicating that these two pathways may serve as core functional modules in *U. pumila* ‘Zhonghua Jinye’ in response to saline–alkali stress and melatonin regulation.

### 2.4. Mining of Core Melatonin-Regulated Genes Based on Mfuzz Clustering Analysis

**Figure 5:**
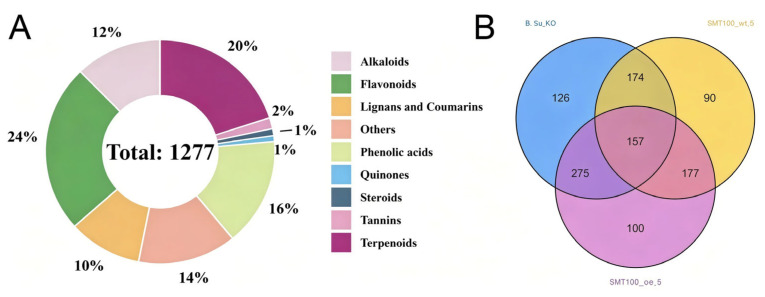
Classification of differential metabolites. Note: (**A**) metabolic substance composition diagram; (**B**) metabolite Venn diagram.

To further clarify the expression dynamics of DEGs, clustering analysis was performed using gene expression data (FPKM) of all genes (Figure 5A). All genes were divided into 12 clusters. In Cluster 2, this module comprised a total of 97 transcription factors (TFs); 41 transcription factors (TFs) were enriched, including AP2 (13, 10.1%), C3H (11, 8.5%), Trihelix (10, 7.7%), bHLH (9, 7.0%), bZIP (8, 6.2%), WRKY (7, 5.4%), GRAS (6, 4.7%), MYB (6, 4.7%), and HB-HD-ZIP (4, 3.1%). The remaining 56 TFs (43.4%) were also strongly induced by both saline–alkali stress and melatonin treatment. The expression heatmap of TFs in Cluster 2 (Figure 5B) showed that AP2, C3H, Trihelix, bHLH, bZIP, GRAS, MYB, and WRKY were induced by saline–alkali stress and melatonin, suggesting their key regulatory roles in saline–alkali stress responses and their potential involvement in melatonin-enhanced stress tolerance. The KEGG enrichment analysis of shared genes among Cluster 2, S_vs_CK, and SMT100_vs_CK revealed significant enrichment of ko00941 flavonoid biosynthesis, ko00199 cytochrome P450, and ko00480 glutathione metabolism pathways.

### 2.5. Screening of Differentially Accumulated Metabolites

Widely targeted metabolomic analysis was conducted using UPLC-MS/MS. A total of 1277 metabolites were identified in positive and negative ion modes. By compound classification (Figure 5A), flavonoids were the most abundant (307, 24.04%), followed by terpenoids (255, 19.97%), phenolic acids (204, 15.98%), tannins (119, 9.32%), and alkaloids (106, 8.30%).

Venn diagrams of differentially accumulated metabolites (DAMs) among groups are shown in Figure 5B. In the S vs. CK group, 732 DAMs were detected, including 488 up-regulated DAMs (e.g., scoparone, plantamajoside, 3-(1,3-benzodioxol-5-yl) acrylaldehyde) and 244 down-regulated DAMs (e.g., solanoic acid I, tenaxigenin I, 1-hydroxy-6-oxocyclohex-2-ene-1-carboxylic acid 1-O-(6″-O-p-coumaroyl) glucoside). In the SMT100 vs. S group, 597 DAMs were detected, including 147 up-regulated DAMs (e.g., myricetin-3-O-(6″-malonyl) glucoside, 13,28-epoxy-11-ursen-3-one, 3′,4′,5,5′,7-pentamethoxyflavone 5-glucoside) and 450 down-regulated DAMs (e.g., 2′-hydroxyisoflavanone, myristicin, machilin). In the SMT100 vs. CK group, 709 DAMs were detected, including 354 up-regulated DAMs (e.g., 5,8,3′-trihydroxy-6,7,4′-trimethoxyflavone 8-O-malonylglucoside, 5,2′,5′-trihydroxy-3,7,4′-trimethoxyflavone 2′-O-β-D-malonylglucoside, 3-(7-methoxy-2H-1,3-benzodioxol-5-yl)acrylaldehyde) and 355 down-regulated DAMs (e.g., 5-hydroxy-4a-methyl-4,4a,5,6,7,8-hexahydronaphthalen-2(3H)-one, 1-O-p-coumaroyl-β-D-glucose, quercetin-4′-O-glucuronide). A total of 157 DAMs were shared among the three comparison groups.

### 2.6. Enrichment Analysis of Differentially Accumulated Metabolites

**Figure 6:**
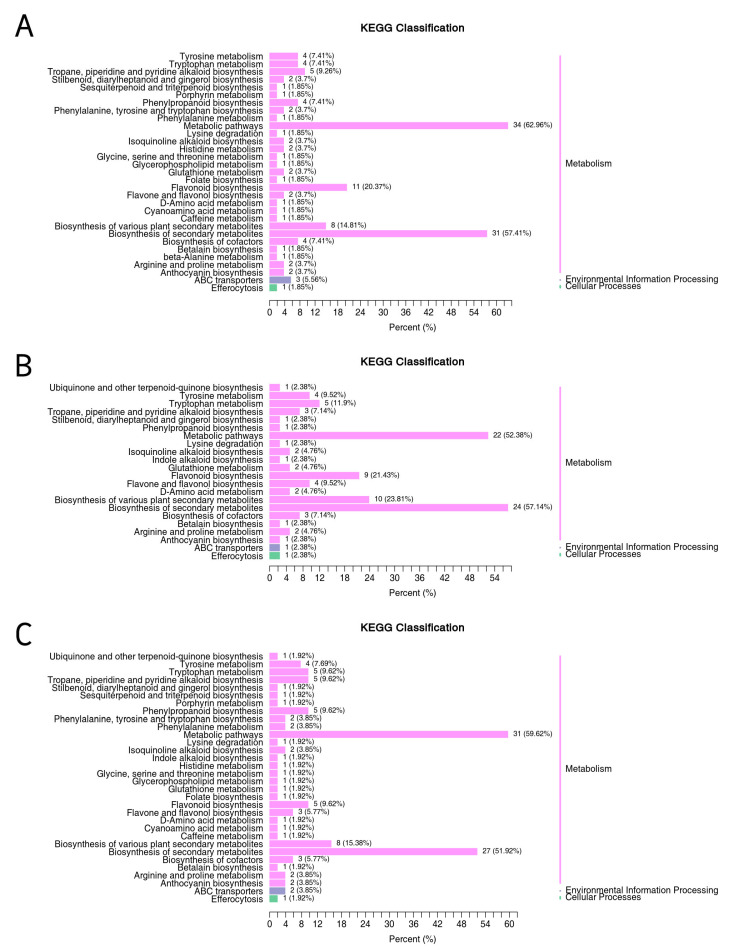
Differential metabolite pathway classification map. Note: (**A**) S vs. CK; (**B**) SMT100 vs. S; (**C**) SMT100 vs. CK.

Differentially accumulated metabolites (DAMs) from the three comparison groups were involved in multiple KEGG pathways (Figure 6A–C). Specifically, DAMs in the S vs. CK group were enriched in 31 pathways (*p* < 0.05), including the biosynthesis of secondary metabolites (31 DAMs) and flavonoid biosynthesis (11 DAMs). DAMs in the SMT100 vs. S group were enriched in 22 pathways (*p* < 0.05), including the biosynthesis of secondary metabolites (24 DAMs) and flavonoid biosynthesis (10 DAMs). DAMs in the SMT100 vs. CK group were enriched in 32 pathways (*p* < 0.05), including the biosynthesis of secondary metabolites (27 DAMs) and flavonoid biosynthesis (8 DAMs). Notably, both the biosynthesis of secondary metabolites and flavonoid biosynthesis pathways were significantly enriched across all comparisons.

### 2.7. Integrated Analysis of Transcriptome and Metabolome

**Figure 7:**
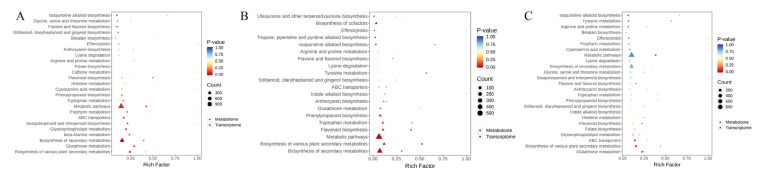
Transcriptome and metabolome association KEGG enrichment analysis *p*-value map. Note: (**A**) S vs. CK; (**B**) SMT100 vs. S; (**C**) SMT100 vs. CK.

Enrichment analysis was performed on differentially expressed genes and differentially accumulated metabolites underlying melatonin-mediated saline–alkali stress responses in *U. pumila* ‘Zhonghua Jinye’ (Figure 7). The results showed that seven pathways were significantly enriched in both transcriptome and metabolome between the S and CK groups, including flavonoid biosynthesis, phenylalanine metabolism, tyrosine and tryptophan biosynthesis, isoquinoline alkaloid biosynthesis, porphyrin metabolism, and cyanoamino acid metabolism.

Between the SMT100 and S groups, seven pathways were significantly enriched in both transcriptome and metabolome, including biosynthesis of secondary metabolites, tyrosine metabolism, flavonoid biosynthesis, isoquinoline alkaloid biosynthesis, tryptophan metabolism, indole alkaloid biosynthesis, and glutathione metabolism.

Between the SMT100 and CK groups, six pathways were consistently enriched in both transcriptome and metabolome, including flavonoid biosynthesis, tyrosine and tryptophan biosynthesis, sesquiterpenoid and triterpenoid biosynthesis, cyanoamino acid metabolism, and porphyrin metabolism.

Notably, flavonoid biosynthesis was the highly conserved metabolic branch in the secondary metabolic network across all three comparison groups. It converts phenylalanine into cinnamoyl-CoA via the phenylpropanoid pathway and ultimately directs carbon flux into flavonoid production. These results indicate that flavonoids play vital roles in the mechanism by which plants alleviate saline–alkali stress.

### 2.8. Analysis of Genes and Metabolites Related to Flavonoid Biosynthesis Pathway

**Figure 8:**
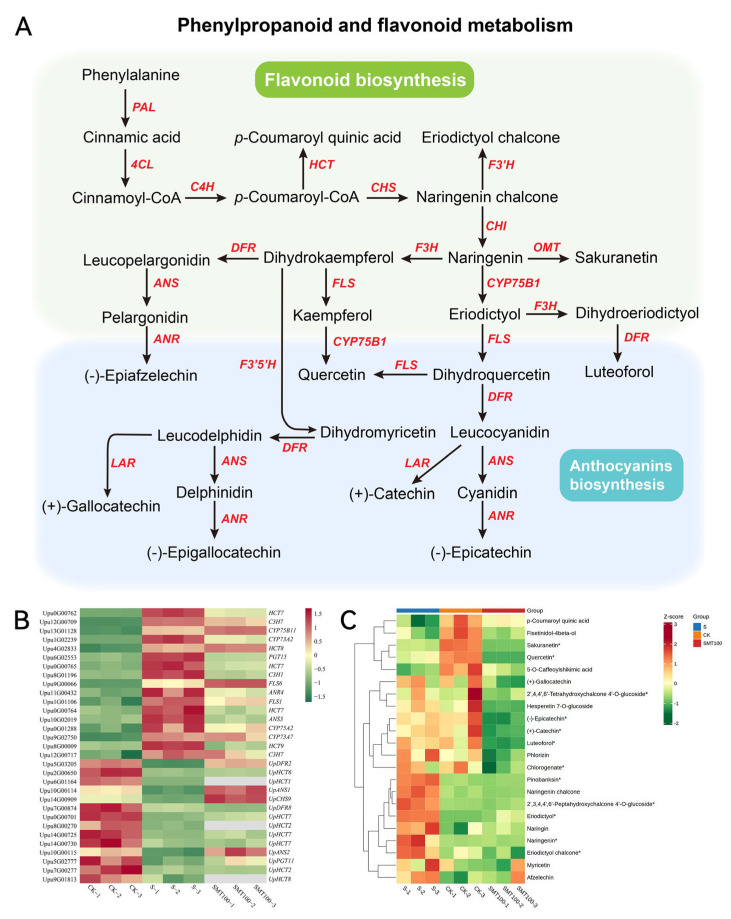
Flavonoid synthesis pathway and differential gene differential metabolite expression heat map. Note: (**A**) flavonoid biosynthetic pathway; (**B**) gene expression heat map; (**C**) metabolite expression heat map.

Integrated transcriptome and widely targeted metabolome analyses were performed to comprehensively characterize the flavonoid biosynthesis pathway regulated by melatonin during saline–alkali stress responses in *U. pumila* ‘Zhonghua Jinye’ (Figure 8). In total, 18 DEGs and seven differentially accumulated metabolites were identified in the flavonoid biosynthesis pathway between the S and CK groups, while 11 DEGs and nine differentially accumulated metabolites were detected between the SMT100 and S groups.

Under saline–alkali stress, the expression levels of *UpHCT1*, *UpANS1*, *UpANS2*, *UpHCT2*, and *UpDFR2* were significantly down-regulated, which inhibited the flavonoid biosynthesis pathway. The modulation of melatonin was accompanied by the accumulation of protective compounds such as sakuranetin, caffeoylshikimic acid, and quercetin, thereby weakening antioxidant capacity and stress defense, and exacerbating stress-induced damage. In contrast, exogenous melatonin treatment specifically up-regulated key structural genes including *UpANS1*, *UpANS2*, *UpDFR2*, *UpCHS9*, and *UpANR7*, which reactivated the flavonoid biosynthesis pathway and promoted the accumulation of flavonoid compounds (e.g., naringenin chalcone, sakuranetin, naringenin, pinocembrin, and quercetin). These changes effectively alleviated saline–alkali stress injury in *U. pumila* ‘Zhonghua Jinye’. These results demonstrate that melatonin confers stress tolerance by coordinately regulating key genes and metabolites in the flavonoid pathway.

Further analysis of gene expression levels revealed that *UpHCT1* was down-regulated approximately 13.88-fold, *UpANS1* by 10.74-fold, and *UpDFR2* by 6.42-fold under saline–alkali stress. During melatonin-mediated regulation, *UpANS1* was up-regulated 17.36-fold, *UpDFR2* by 5.55-fold, *UpCHS6* by 1.78-fold, and *UpLAR2* by 2.10-fold. *UpANS1* exhibited the most dramatic expression changes in response to both saline–alkali stress and melatonin application: it was sharply repressed by stress but strongly restored by melatonin. These findings indicate that *UpANS1* may serve as a core hub gene in saline–alkali stress responses and melatonin-mediated stress tolerance in *U. pumila* ‘Zhonghua Jinye’.

### 2.9. qRT-PCR Validation of Key Differentially Expressed Genes

**Figure 9:**
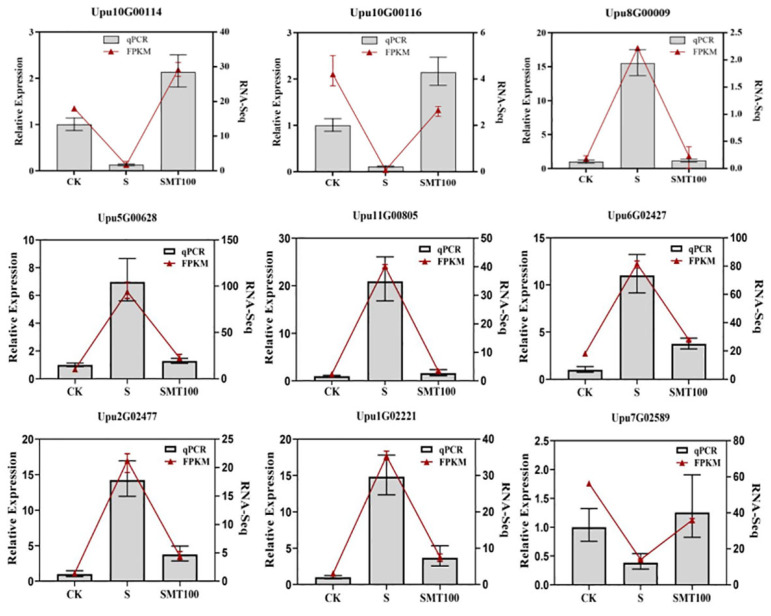
qRT-PCR verification of expression level of 9 DEGs in RNA sequencing. Note: Column represents the expression level of differential genes, error bar represents the standard deviation between biological replicates, and broken line represents the FPKM value of transcriptome.

qRT-PCR was performed to verify the expression patterns of nine key DEGs involved in flavonoid biosynthesis, hormone signal transduction, and antioxidant systems across all samples (Figure 9). The expression trends of the nine genes obtained by qRT-PCR were consistent with those obtained by RNA-seq, confirming the reliability of the transcriptomic profiling.

## 3. Discussion

### 3.1. Exogenous Melatonin Alleviates Physiological Damage of Plants Under Saline–Alkali Stress

Saline–alkali stress is one of the main abiotic stresses that limit plant growth. Compared with single salt stress, a saline–alkali complex environment will not only lead to ion toxicity, but also further destroy the water state and metabolic balance of plant cells [22,23]. Therefore, plants need to maintain physiological homeostasis through multi-level regulatory mechanisms. In recent years, a large number of studies have shown that melatonin plays an important role in plant tolerance to saline–alkali stress [24,25].

In terms of the physiological and biochemical aspects, with the prolongation of saline–alkali stress time, in the leaves of *U. pumila* ‘Zhonghua Jinye’, antioxidant enzyme activity is one of the important factors of saline–alkali stress. When plants are stressed, the level of reactive oxygen species in plants usually increases significantly, and the excessive accumulation of ROS will destroy the cell membrane structure and cause oxidative stress. In order to remove excessive ROS, plants will initiate antioxidant enzyme systems to reduce oxidative damage [26]. As shown in Figure 1, under the treatment of spraying melatonin, the activity of antioxidant enzymes increased to varying degrees, while the content of H_2_O_2_ and O_2^−^_ decreased. It can be seen from this study that melatonin mitigates saline–alkali stress in *U. pumila* ‘Zhonghua Jinye’ by bolstering the antioxidant enzyme system to suppress ROS accumulation, thereby alleviating membrane lipid peroxidation.

### 3.2. Response of Genes and Metabolites in Flavonoid Biosynthesis Pathway Regulated by Melatonin

Flavonoids are an important part of the non-enzymatic antioxidant system in plants, which can directly scavenge ROS, chelate metal ions, and stabilize cell membrane structure [27,28]. The enhanced accumulation of flavonoids induced by melatonin is directly related to the reduction in oxidative damage in physiological indicators, which constitutes a complete pathway from signal perception to gene regulation, then to metabolite synthesis, and finally to antioxidant [29,30,31]. This finding provides a new perspective for understanding how melatonin enhances stress resistance by reprogramming secondary metabolism. Similarly, in soybean under drought stress and *Cyclocarya paliurus* under salt stress, the promotion of melatonin on flavonoid synthesis was also observed, indicating that this may be a conservative mechanism for melatonin to exert its stress resistance function [32,33,34]. For example, the MYB-bHLH-WD40 complex directly regulates the expression of genes such as ANS to control anthocyanin synthesis [35,36]. In summary, this study provides a working model suggesting that melatonin enhances saline–alkali tolerance in *U. pumila* by modulating the flavonoid biosynthetic pathway, likely through the putative regulation of key genes such as *UpANS1*.

This study, through a combined transcriptome and metabolome analysis, systematically suggests that the ‘flavonoid biosynthesis’ pathway is the core regulatory target of melatonin to alleviate saline–alkali stress in woody plants. Saline–alkali stress led to the down-regulation of several key genes in this pathway (such as *UpANS1*, *UpANS2*, *UpDFR2*, *UpHCT1*) and reduced the accumulation of downstream flavonoid metabolites. This may be a strategy for plants to inhibit secondary metabolism in order to save energy under severe stress, but it also weakens their chemical defense capabilities [37,38]. Exogenous melatonin treatment reversed this trend, significantly up-regulated the expression of a series of genes from *UpCHS1* to *UpANS1*, and promoted the synthesis and accumulation of flavonoids.

## 4. Conclusions

In this study, the molecular mechanism of exogenous melatonin alleviating saline–alkali stress in *U. pumila* ‘Zhonghua Jinye’ was systematically analyzed by integrating physiology, transcriptomics and metabolomics analysis. Melatonin 100 μmol·L^−1^ treatment can effectively alleviate the oxidative damage caused by saline–alkali stress by significantly enhancing the antioxidant enzyme (SOD, CAT, APX, GR) system.

Through transcriptome and metabolome data, it was revealed that the flavonoid biosynthesis pathway plays a key role in melatonin regulation in *U. pumila* ‘Zhonghua Jinye’ in response to saline–alkali stress. Saline–alkali stress inhibited the expression of key genes such as *UpANS1*, *UpANS2*, *UpHCT1*, and *UpDFR2* in this pathway, and reduced the accumulation of flavonoids such as quercetin and kaempferol. Exogenous melatonin treatment can significantly up-regulate the expression of *UpANS1*, *UpANS2*, *UpDFR2*, *UpCHS1*, *UpF3H6*, *UpLAR2* and other genes, re-activate the flavonoid synthesis pathway, and promote the synthesis of flavonoids with antioxidant activity, thereby enhancing the overall antioxidant capacity of the plant. The data support a strong association between melatonin application, enhanced antioxidant enzyme activities, and the reprogramming of the flavonoid biosynthetic pathway. We propose a working model wherein melatonin modulates the expression of key structural genes (e.g., *UpANS1*) and associated transcription factors. However, this study represents an association-based analysis; further functional validation is required to fully elucidate the complete regulatory network.

This study clarified the complete regulatory network of melatonin in woody plants to enhance saline–alkali tolerance by activating the flavonoid biosynthesis pathway, which not only provides a new approach to understanding the plant stress resistance function of melatonin, but also lays a theoretical foundation for improving the application of *U. pumila* resistance to abiotic stress.

## 5. Materials and Methods

### 5.1. Plant Materials and Cultivation

Two-year-old cuttings of *U. Pumila* ‘Zhonghua Jinye’ were used as experimental materials. The experiment was conducted in the research greenhouse of Hebei Academy of Forestry and Grassland Sciences (coordinates: 38.14209473° N, 114.47560635° E). Environmental conditions were set as follows: day/night temperature of 25C/18 °C, photoperiod of 12 h·d^−1^ (light intensity 720 µmol·m^−2^·s^−1^), and relative humidity of 65%. Healthy and uniform seedlings were selected and transplanted into 3.0 L plastic pots (15 cm diameter × 20 cm height, and one seedling per pot) filled with a mixed substrate of garden soil and peat soil at a volume ratio of 1:1. During the acclimatization period, half-strength Hoagland nutrient solution was irrigated every 5 days. After 30 days of cultivation, seedlings with consistent growth were selected for subsequent stress treatments.

### 5.2. Melatonin Treatment

Test reagent: Melatonin (MT, Sigma-Aldrich, Cat. No. M5250, purity ≥ 99%, synthetic, St. Louis, MO, USA). MT was initially dissolved in a small amount of anhydrous ethanol, and then diluted to the required concentrations with deionized water containing 0.05% (*v*/*v*) Tween-20. All treatment solutions, including the Control (CK) and Saline–alkali (S) groups, contained 0.1% (*v*/*v*) absolute ethanol and 0.05% (*v*/*v*) Tween-20 to exclude solvent effects. The experiment adopted a completely random design of potting method. Based on the ion composition characteristics of coastal saline–alkali soil in China, the compound saline–alkali solution was prepared with a molar ratio of NaCl: Na_2_CO_3_: NaHCO_3_: Na_2_SO_4_ = 21:3:4:1 and a stable pH value of 8.8. The experiment was formally initiated on 10 June 2024. To prevent instantaneous seedling damage caused by salt shock under high-concentration stress, a gradient stress treatment method was adopted. The treatment started with an initial concentration of 50 mmol·L^−1^, which was increased by 50 mmol·L^−1^ every two days until the preset concentration of 150 mmol·L^−1^ was reached, after which the formal stress duration was recorded. During the treatment period, each pot was irrigated with 200 mL of the corresponding saline–alkali solution every two days. The exudate solution in the tray was poured back into the pot in a timely manner to maintain stable substrate salinity, and three biological replicates were set for each treatment. After the saline–alkali stress was stable, the seedling leaves were evenly sprayed with different concentrations of exogenous melatonin solution. Four concentrations of melatonin were set up, with three replications for each treatment (Table 2). Starting from 10 June 2024, seedlings were firstly treated with 150 mmol·L^−1^ saline–alkali solution. Then, exogenous melatonin solutions of different concentrations were sprayed onto the plant leaves until the liquid was about to drip from the leaf tips. The spraying was conducted every 2 days for a total of 28 days. Each treatment contained 5 pots with three biological replicates, with 25 pots in total.

At 0, 14, and 28 days of treatment, the 3rd to 5th fully expanded functional leaves from the top of three randomly selected plants per treatment were collected for the determination of physiological indices. Samples for physiological indices were immediately frozen in liquid nitrogen and stored in an ultra-low temperature freezer at −80 °C until analysis to prevent enzymatic degradation. For transcriptome and metabolome analyses, leaf samples from the CK, S, and SMT100 groups were harvested at 28 days of treatment, immediately frozen in liquid nitrogen, and stored at −80 °C.

### 5.3. Determination of Physiological Indices

Enzyme activity was calculated based on the standard curve provided and normalized to the fresh weight (FW) of the tissue. Antioxidant enzyme activities superoxide dismutase (SOD), catalase (CAT), ascorbate peroxidase (APX), and glutathione reductase (GR)) and reactive oxygen species (ROS) levels (hydrogen peroxide and superoxide anion) were assessed in *U. pumila* ‘Zhonghua Jinye’ leaves exposed to varying saline–alkali concentrations. Both enzymatic activities and ROS contents were quantified using commercial spectrophotometric and micro-detection kits, respectively (Solarbio, Beijing, China) Sampling was conducted at 14-day intervals, with three biological replicates per treatment. Plant tissues (0.1 g) were homogenized in 1 mL of ice-cold extraction buffer using a mortar and pestle. The homogenate was centrifuged at 12,000× *g* for 15 min at 4 °C. Supernatants were collected for assays according to the manufacturer’s instructions.

### 5.4. Data Statistics and Analysis

Microsoft Excel was used for data sorting, preprocessing and figure plotting. All data were presented as means ± standard error (SE). Two-way analysis of variance (ANOVA), correlation analysis and principal component analysis (PCA) were performed using SPSS 27.0. The subordinate function method was adopted for comprehensive evaluation, and the least significant difference (LSD) test was used for multiple comparisons and significance analysis.

### 5.5. Transcriptome and Metabolome Sequencing

Based on preliminary physiological analysis, the SMT100 treatment performed significantly better than other melatonin concentrations in terms of ROS scavenging and antioxidant enzyme activities. To further elucidate the molecular mechanism by which melatonin alleviates saline–alkali stress, three groups with the most significant phenotypic and physiological differences were selected for transcriptome sequencing: control group (CK), S and SMT100.

#### 5.5.1. RNA Isolation, Library Construction and Transcriptome Sequencing

Total RNA was extracted from leaf samples using a Plant Total RNA Isolation Kit (Vazyme, Nanjing, China) following the manufacturer’s instructions for polysaccharide- and polyphenol-rich tissues. RNA concentration, purity, and integrity were verified using a nucleic acid–protein detector and agarose gel electrophoresis. High-quality RNA was used to construct cDNA libraries for RNA-seq. After quality validation using an Agilent 2100 Bioanalyzer (Agilent Technologies, Santa Clara, CA, USA) and Agilent High Sensitivity DNA Kit (Cat. No. 5067-4626, Agilent Technologies, USA), the qualified libraries were sent to Metware Biotechnology Co. (Woburn, MA, USA), for high-throughput sequencing.

#### 5.5.2. Transcriptome Data Analysis

Raw sequencing data were subjected to quality control and filtering using Fastp software (v0.23.4, OpenGene) to obtain high-quality clean reads. Clean reads were mapped to the reference genome of *Ulmus pumila* (GenBank version: GCA_028858055.1) with HISAT2 (v2.2.1). Gene expression levels were calculated as FPKM values using StringTie. Differential expression analysis was performed using the DESeq2 (v1.34.0) R package with thresholds of |log_2_ Fold Change| ≥ 1 and adjusted *p*adj < 0.05 to identify differentially expressed genes (DEGs). Gene Ontology (GO) functional annotation and Kyoto Encyclopedia of Genes and Genomes (KEGG) pathway enrichment analysis of DEGs were conducted using the clusterProfiler R package. Soft clustering of gene expression trends was carried out using the Mfuzz R package.

#### 5.5.3. Metabolite Extraction and Targeted Metabolomic Analysis

Metabolomics was performed to identify potential metabolites involved in the response to melatonin treatment. Three groups (CK, S, SMT100) with three biological replicates each (total nine samples) were vacuum freeze-dried for 63 h and ground using a mixer mill (MM400, Retsch, Haan, Germany) for 1.5 min. Then, 30 mg of lyophilized powder was extracted with 1500 μL of pre-cooled (−20 °C) 70% methanol aqueous solution. After centrifugation at 12,000 rpm for 3 min, the supernatant was filtered through a 0.22 μm membrane and stored in sample vials for UPLC-MS/MS analysis.

An ultra-performance liquid chromatography (UPLC) coupled with tandem mass spectrometry (MS/MS) system was used for data acquisition. Chromatographic conditions: Waters ACQUITY UPLC HSS T3 column (1.8 μm, 2.1 mm × 100 mm); mobile phase A was 0.1% formic acid in water, and mobile phase B was 0.1% formic acid in acetonitrile; gradient elution was applied. Mass spectrometry conditions: AB Sciex QTRAP 650+ mass spectrometer with an electrospray ionization (ESI) source, operated in both positive and negative ion modes (AB Sciex, Marlborough, MA, USA). Metabolites were identified based on the self-built MWDB database (Metware Biotechnology), and quantified using the multiple reaction monitoring (MRM) mode.

#### 5.5.4. Metabolite Data Analysis

Multivariate statistical analysis was performed using OPLS-DA. The model’s robustness was validated by R2Y (0.98) and Q2 (0.92) values, along with a permutation test (200 iterations) where the intercept of the Q2 regression line was negative (−0.15), indicating no overfitting. Differentially accumulated metabolites (DAMs) were screened using the criteria of VIP > 1.0, |log2 Fold Change| ≥ 1, and adjusted *p*adj < 0.05 (based on FDR correction). KEGG pathway enrichment analysis was performed using the MetaboAnalyst 5.0 platform. Metabolites in specific pathways were subjected to Z-score normalization and used to generate heatmaps.

#### 5.5.5. Combined Transcriptomic and Metabolomic Analysis

KEGG annotation results of DEGs from transcriptome and DAMs from metabolome were integrated. Joint-pathway enrichment analysis was conducted using the Joint-Pathway Analysis function in MetaboAnalyst 5.0. Co-enriched pathways were selected to construct integrated pathway maps, showing synergistic changes in gene expression and metabolite accumulation.

#### 5.5.6. qRT-PCR Validation

Nine differentially expressed genes were selected from the transcriptome to verify the reliability of transcriptomic data using real-time quantitative PCR (RT-qPCR). Total RNA was reverse-transcribed into cDNA using a reverse transcription kit. Primers were designed using Primer 3 based on CDS sequences from transcriptome sequencing, with the *Upu11G00853* gene as the internal reference; primer information is listed in Table 3. RT-qPCR was performed on a Bio-Rad CFX Manager system (Bio-Rad, Hercules, CA, USA). The amplification program consisted of 95 °C for 3 min, followed by 40 cycles of 95 °C for 10 s and 60 °C for 30 s. A melt curve analysis (65 °C to 95 °C) was performed to verify amplicon specificity. The 10 μL reaction mixture consisted of 5 μL of 2x SG Green qPCR Mix, 0.5 μL each of Primer F/R (10 μM), 1 μL of cDNA template, and 3 μL of nuclease-free water. Each sample was run in triplicate (technical replicates). Relative expression levels were calculated using the 2^−ΔΔCt^ method.

## Figures and Tables

**Table 1:** The sample comparison results.

Sample	Clean Reads	Mapped Reads (%)	Q30%	GC%
CK-1	73,385,228	63,846,407 (87.00)	97.43	45.74
CK-2	78,851,836	68,674,658 (87.09)	97.34	45.55
CK-3	67,492,780	58,819,225 (87.15)	97.35	45.59
S-1	45,116,134	38,490,871 (85.32)	96.11	45.39
S-2	46,840,442	40,072,102 (85.55)	95.84	45.42
S-3	44,858,526	38,337,208 (85.46)	95.95	45.39
SMT100-1	47,463,390	40,793,810 (85.95)	96.07	45.62
SMT100-2	47,877,928	40,999,516 (85.63)	95.93	45.65
SMT100-3	50,693,792	42,950,751 (84.73)	96.46	45.81

**Table 2:** Concentration of treatment solution.

Treatment Solution	Treatment Group
0 mmol·L^−1^ saline–alkaline solution + 0 μmol·L^−1^ MT	CK
150 mmol·L^−1^ saline–alkaline solution + 0 μmol·L^−1^ MT	S
150 mmol·L^−1^ saline–alkaline solution + 50 μmol·L^−1^ MT	SMT50
150 mmol·L^−1^ saline–alkaline solution + 100 μmol·L^−1^ MT	SMT100
150 mmol·L^−1^ saline–alkaline solution + 200 μmol·L^−1^ MT	SMT200
150 mmol·L^−1^ saline–alkaline solution + 400 μmol·L^−1^ MT	SMT400

**Table 3:** RT-qPCR primers sequences.

Gene ID	Primer Name	Primer Sequences
*Upu11G00853*	648-P16-F_Upu11G00853	CCGTGACATGAAGGAGAA
	648-P16-R_Upu11G00853	GAGATGGTTGGAAGAGGA
*Upu10G00114*	648-P30-F_Upu10G00114	AACGACATAGATTCGGAAGACC
	648-P30-R_Upu10G00114	TCCTCAATGGGAAGCTCAAA
*Upu10G00116*	648-P28-F_Upu10G00116	ATTGGGATTGGAAGAAGATAGG
	648-P28-R_Upu10G00116	TGTTGTGGAGGATGAAAGTGAG
*Upu8G00009*	648-P27-F_Upu8G00009	CAGGGTGAGAAAGACGACA
	648-P27-R_Upu8G00009	ACGAGGACCTTGCTAAGTG
*Upu5G00628*	648-P9-F_Upu5G00628	CAGGCAGTGGGTAGTAGTGG
	648-P9-R_Upu5G00628	ATTGACAGGGTATTTGGAGC
*Upu11G00805*	648-P13-F_Upu11G00805	TGAGTTGACGGTTAGGATT
	648-P13-R_Upu11G00805	GTTGGAGAACGACAAGGAG
*Upu6G02427*	648-P22-F_Upu6G02427	GATGGATGGGTGTTTTGGT
	648-P22-R_Upu6G02427	GGTCTTTGGGGCTTATGAT
*Upu2G02477*	648-P8-F_Upu2G02477	CAAATCTCCACTGCTCCCT
	648-P8-R_Upu2G02477	TGCTTCTTGTTCTACCCCA
*Upu1G02221*	648-P19-F_Upu1G02221	AATCTCCACTGCTCCCTTC
	648-P19-R_Upu1G02221	CTTCCCTGCCTCTTGTTCT
*Upu7G02589*	648-P17-F_Upu7G02589	CTGCCTTCCTCTTCCCTTA
	648-P17-R_Upu7G02589	TCCCCAATCACCCATCTTT

## Data Availability

The original contributions presented in this study are included in the article. Further inquiries can be directed to the corresponding authors.
